# Innate Immunity and Breast Milk

**DOI:** 10.3389/fimmu.2017.00584

**Published:** 2017-05-29

**Authors:** Nicole Theresa Cacho, Robert M. Lawrence

**Affiliations:** ^1^Division of Neonatology, Department of Pediatrics, University of Florida, Gainesville, FL, United States; ^2^Division of Pediatric Infectious Disease, Department of Pediatrics, University of Florida, Gainesville, FL, United States

**Keywords:** human milk, breast milk, innate immunity, colostrum, preterm

## Abstract

Human milk is a dynamic source of nutrients and bioactive factors; unique in providing for the human infant’s optimal growth and development. The growing infant’s immune system has a number of developmental immune deficiencies placing the infant at increased risk of infection. This review focuses on how human milk directly contributes to the infant’s innate immunity. Remarkable new findings clarify the multifunctional nature of human milk bioactive components. New research techniques have expanded our understanding of the potential for human milk’s effect on the infant that will never be possible with milk formulas. Human milk microbiome directly shapes the infant’s intestinal microbiome, while the human milk oligosaccharides drive the growth of these microbes within the gut. New techniques such as genomics, metabolomics, proteomics, and glycomics are being used to describe this symbiotic relationship. An expanded role for antimicrobial proteins/peptides within human milk in innate immune protection is described. The unique milieu of enhanced immune protection with diminished inflammation results from a complex interaction of anti-inflammatory and antioxidative factors provided by human milk to the intestine. New data support the concept of mucosal-associated lymphoid tissue and its contribution to the cellular content of human milk. Human milk stem cells (hMSCs) have recently been discovered. Their direct role in the infant for repair and regeneration is being investigated. The existence of these hMSCs could prove to be an easily harvested source of multilineage stem cells for the study of cancer and tissue regeneration. As the infant’s gastrointestinal tract and immune system develop, there is a comparable transition in human milk over time to provide fewer immune factors and more calories and nutrients for growth. Each of these new findings opens the door to future studies of human milk and its effect on the innate immune system and the developing infant.

## Introduction

The innate immune system is the first line of defense against infection and is activated within minutes, reacting in a nonspecific, preprogrammed, and patterned manner to various infectious or foreign (non-self) stimuli (1). The infant’s immune system is immature at birth, and this immaturity is pronounced for the premature infant placing the infant at increased risk of infection (2). Important developmental immune deficiencies at birth include incomplete physical and chemical barriers, poor innate effector cell function, limited and delayed secretory immunoglobulin A (IgA) production, incomplete complement cascade function, and insufficient anti-inflammatory mechanisms of the respiratory and gastrointestinal (GI) tracts.

Human milk is the everchanging secretions of the human breast, an evolving composition of nutrients and active factors. Just as nutrition and protection of the fetus occurs through the mutable nature of the uterus, placenta, and amniotic fluid, the evolution of human milk from colostrum through transitional milk to mature milk provides nutrition and protection appropriate for the time-affected development of the infant (3). There is a large body of evidence documenting the benefits of human breast milk for human infants, in diminishing morbidity and mortality and protecting against specific infections during the period of breastfeeding (4–6). Additional data demonstrate long-term health benefits for the infant (and the mother) beyond the period of lactation (7, 8) and led to the current recommendations for duration of exclusive breastfeeding from the American Academy of Pediatrics (9) and the World Health Organization. Although research into the specific factors in human breast milk, which lead to the remarkable health benefits of exclusive breastfeeding, has been ongoing for decades; there are still intriguing mysteries of how human milk contributes to the development and regulation of both the infant’s innate (10) and adaptive immune function (11–13).

**Figure 1:**
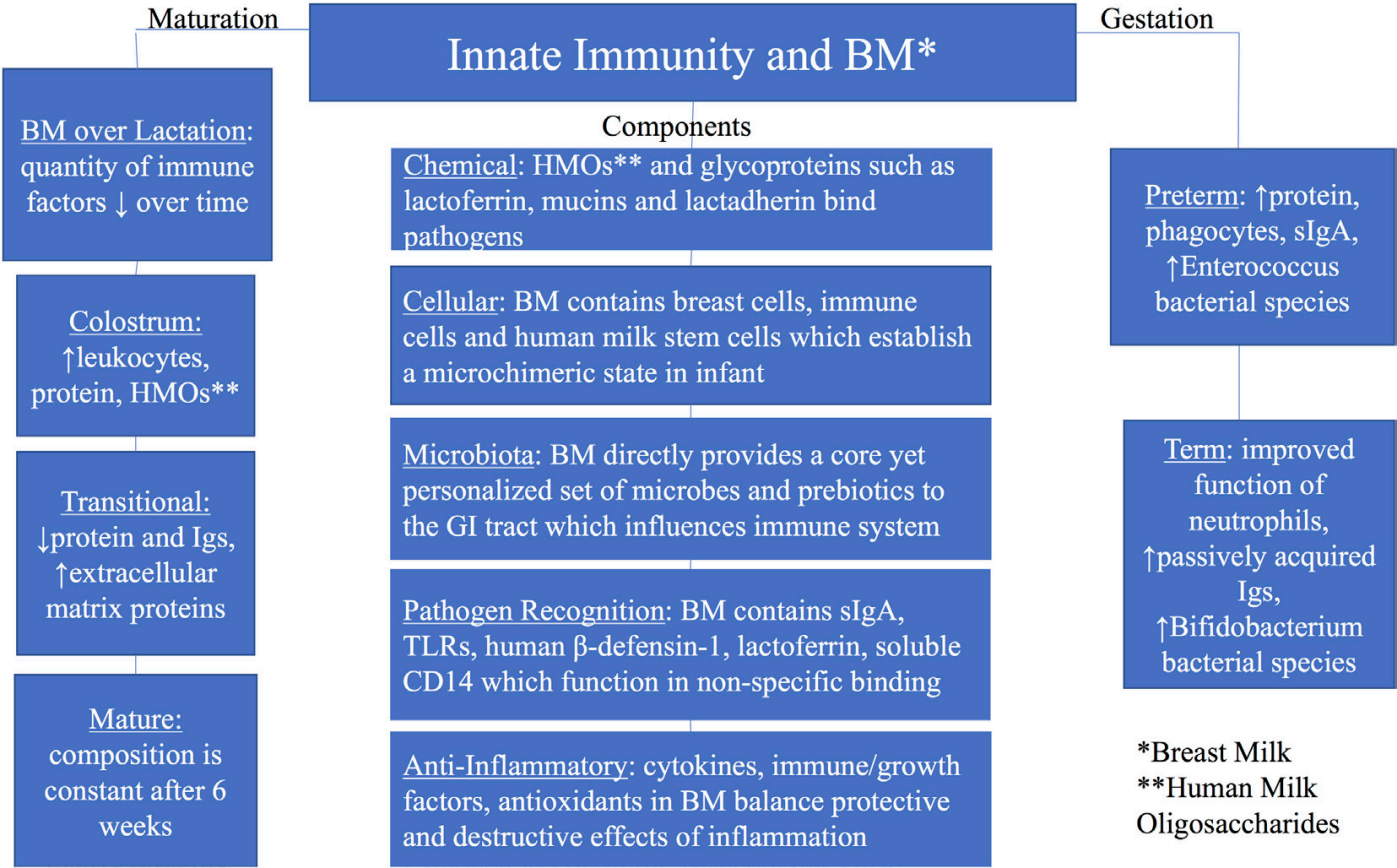
**Summary of innate immunity and breast milk with groups including components of the innate immunity, maturation of breast milk over lactation, and breast milk by gestation (preterm and term)**.

Elucidating the relationship between the innate immune system and human milk, as well as their individual and interactive transition over time, remains challenging. Many components in milk are multifunctional, serving as enzymes, antimicrobial proteins/peptides (AMPs), growth factors, chemokines, antioxidants, anti-inflammatory elements, prebiotics, probiotics, and nutrients for the growing infant (14, 15). The use of modern molecular approaches such as microbial genomics, metabolomics, proteomics, and glycomics has led to novel discoveries in both composition and function of human milk components. This review will focus on the current understanding of the critical interactions between human breast milk and the infant’s developing innate immune system (Figure 1) (12, 16–18).

## Chemical Barriers of Innate Immunity

The chemical barrier of the intestine is predominately the mucus layers lining the GI tract. These mucus layers minimize antigenic contact between epithelial cells and commensal bacteria as well as potentially pathogenic bacteria. Antimicrobial peptides produced by Paneth cells, released into the mucus layer, bolster this chemical barrier innate effect by neutralizing microbes *via* various mechanisms. The multifunctionality of individual human milk factors adds another layer of complexity to the innate protection effected within the intestinal mucus layers.

Human milk oligosaccharides (HMOs) are the predominant glycans and important nutrients in human milk. They function in direct pathogen binding and as prebiotics facilitating the establishment of a healthy infant microbiome (2). The human milk glycoproteins (HMGPs) vary in size, structure, and amount in human milk and can be classified based on their location relative to the cell (secreted or attached to the cell membrane) and the different mucus layers. Mucin 1 (MUC1) and Mucin 4 (MUC4), gangliosides (GM1, GM3, and GD3), and glycoproteins similarly function by binding pathogens and do so without stimulating an inflammatory response (19). MUC1 and MUC4 have been described as binding to specific pathogens including HIV, rotavirus, *Escherichia coli*, and *Salmonella*. Other HMGPs have demonstrated binding to *H. influenzae*, Streptococci, *Helicobacter pylori*, Reovirus, *E. coli*, and *Burkholderia cepacia*. The specific molecular mechanism of this binding by glycans and interference with infection by the pathogen requires additional clarification. Unraveling the complex glycoprotein–ligand interactions will require application of newer technologies such as nanosurface plasmon resonance and glycan microarrays (20). Another challenge is to demonstrate whether, how, and when this occurs in a mother–infant dyad exposed to a specific pathogen through changes in the glycan composition of the mother’s milk (21).

Lactoferrin (LF), another glycoprotein abundant in colostrum and transitional milk and ubiquitously expressed in most exocrine secretions, is one of the best studied glycoproteins in human milk. LF has multiple functions in host defense through binding iron, binding to bacterial membranes, inhibition of tumor necrosis factor-alpha (TNF-α) and interleukin-1β (IL-1β), stimulating the activity, maturation of lymphocytes, and contributing to an anti-oxidizing mileau (22). Peptide breakdown products of LF, lactoferricin and lactoferrampin, have specific direct antibacterial and antifungal effects (23).

There are numerous other HMGPs that have been described but the extent and specificity of their immune function need to be elucidated. Lactadherin can inactivate viruses and limits inflammation by increasing the effective phagocytosis of apoptotic cells. The sialic acid component of lactadherin seems to directly interact with rotavirus while the protein backbone of the molecule demonstrates a proangiogenic effect on neovascularization. The list of bioactive glycoproteins in human milk is still expanding, and their individual multifunctional nature is just being described. Butyrophilin, leptin, adiponectin, bile salt-stimulated lipase lysozyme, lactoperoxidase (LP), xanthine dehydrogenase, α-lactalbumin, κ-casein, and β-casein are just a few of these glycoproteins requiring additional study (19).

## Cellular Contributors to Innate Immunity

The cellular layers of the infant’s intestine include the barrier of epithelial cells with the tight junctions, specialized goblet, Paneth and microfold cells, the lamina propria and Peyer’s patches with macrophages, neutrophils, and dendritic cells. The immunity provided by these cellular interactions is partially innate and partially the beginning of adaptive immunity (24). Immaturity of the intestinal barrier function, limitations in production of AMPs, and ineffective response and action of epithelial cells and phagocytes place the infant at risk for infection in the neonatal period (25, 26).

Within fresh human milk, there is a remarkable repertoire of heterogeneous living cells. Cells originating in the breast include lactocytes (secretory cells), myoepithelial cells from the ducts as well as progenitor cells and mammary stem cells, and a small number of squamous epithelial cells from the nipple and skin of the breast. Cells originating from the blood, which are in human milk, include immune cells (macrophages, neutrophils, and lymphocytes), hematopoietic progenitor cells, and hematopoietic stem cells (27, 28). The role of live breast cells in human milk in the infant remains uncertain. New theories are being formulated to explain the fact that these breast milk epithelial cells are capable of motility and can in primary culture form functioning mammospheres (29, 30). Hassiotou et al. (29) explain the fact that these cells are in different stages of differentiation as a continuum of mammary development, albeit that does not answer the question why they are in such large numbers in expressed human breast milk.

There are large numbers of macrophages present in early lactation that decrease with the maturation of the milk (31, 32). These macrophages appear to function by phagocytizing pathogens without initiating a significant, unregulated inflammatory response. An increase in milk leukocytes, above the number usually present at the specific stage of lactation, occurs with infection in either the mother or the infant and suggests a functional role for milk leukocytes in protecting the infant (21, 33, 34). Breast milk from mothers of infants with severe bronchiolitis demonstrated an increased number of live cells. Those live cells produced a specific cytokine profile response when stimulated with live respiratory syncytial virus, a common cause of bronchiolitis (35). These data directly support the concept of mucosal-associated lymphoid tissue and that cells and/or activating factors are transferred from the mother in her breast milk to the infant at the time of infection or exposure to an infection. Additional studies measuring changes in the bioactive factors in human milk of mothers with sick infants or who are sick themselves for various specific infections should provide more insight into the essential bioactive factors in human milk for protection against specific infections.

The human milk stem cells (hMSCs) likely play an important role in the “regeneration” of the breast in preparation for lactation and theoretically in the infant’s tolerance of maternal cell antigens as these cells from human milk do establish a microchimeric state in the infant. Hassiotou et al. (29) have demonstrated the pleuripotential differentiation nature of hMSCs. hMSCs were able to differentiate into the three germ layer cell lineages, in *in vitro* testing. Expression of octamer-binding transcription factor 4, related to “self-renewal” functions, is upregulated in hMSCs isolated from human milk (33). Using milk from genetically modified mice, Hassiotou et al. (36) demonstrated the persistence of modified milk stem cells within the brain, thymus, pancreas, liver, spleen, and kidneys of non-modified mice (36). These data specifically suggest a potential role for hMSCs in tissue regeneration in the breastfed infant and perhaps regeneration of cells of the infant’s innate immune system. Beyond that, the hMSCs in human milk may provide a ready source of patient-specific stem cells with a true multilineage potential for the study of such stem cells and variables related to breast cancer, tissue regeneration, and even bioengineering.

## Microbiota

In the past, human milk was considered sterile, but that is far from the truth. Using culture techniques, the majority of bacteria identified as facultative anaerobes in human milk belong to the *Staphylococcus* and *Streptococcus* species and other species in smaller numbers (*Propionibacterium, Rothia, Enterococcus*, and *Lactobacillus* species) (37, 38). Obligate anaerobic bacteria were later identified, *Bifidobacterium* and *Bacteroides* species (39–41). Culture-independent techniques (sequencing and metagenomics analysis) have demonstrated a significantly more complex and diverse group of bacteria in human milk (42, 43). There is a large degree of interindividual variability in the milk microbiome. Similar factors affecting the infant’s or the mother’s intestinal microbiomes also affect the milk microbiome (genetics, mode of delivery, geographic area, gestational age, maternal diet and nutrition, antibiotics, lactation stage, etc.) (42, 44). Nevertheless, most samples from healthy women appear to contain a “core” microbiome (34, 45). Hunt et al. name nine bacterial groups and Jimenez et al. reported seven bacterial groups. These two analyses shared just three common bacterial groups: *Staphylococcus, Streptococcus*, and *Propionibacterium* (34, 45). Variability between these two studies and others (43) can be attributed to the use of different primers, types of sequencing, comparison with different microbial reference libraries, possible variation due to geographic regions, and timing of the milk collection as well as technique and sterility of milk collection (43, 46, 47).

The actual origin of the milk microbiota remains uncertain (48). Postulated origins include mammary gland itself, skin flora of the breast, an “entero-mammary pathway” where intestinal bacteria translocate and home to the breast and retrograde flow from the infant’s mouth into the breast (12, 49–51). Tracing the origins of milk microbiota and the relative influence of environmental factors on its makeup should inform our understanding of the role of the common groups of bacteria or their interaction in symbiosis with the infant’s innate immune system and developing intestine.

Human milk directly contributes to the establishment of the intestinal microbiota and facilitates a symbiosis between that microbiota and the infant by providing essential nutrients, in particular milk glycans or HMOs, for microbial metabolism (52). Nanthakumar et al. (53) proposed that specific microbiota colonizing the gut early on in the infant induce the expression of intestinal epithelial cell (IEC) fucosyltransferase 2. This leads to fucosylation of surface markers on GI epithelial cells, which enhances the growth of microbes utilizing fucose as part of their metabolism (53). Microorganisms utilize the available glycosaminoglycans, glycoproteins, glycolipids, and oligosaccharides (milk glycans) as prebiotics to facilitate growth (54). The intestinal microbiota limits the growth of pathogenic bacteria by competition for nutrients and receptors. Specific microbes facilitate the formation of the intestinal mucus layer and the development of the IEC barrier and submucosal lymphoid structures. Separately, the HMOs bind to surface molecules of bacteria and viruses preventing binding to the intestinal epithelium and appear to diminish intestinal inflammation *via* signaling pathways (54). Newburg and Morelli (55) describe the symbiosis of the microbiota and the infant’s intestinal development as dependent on HMOs in the mother’s milk. The “commensal” microbes, specifically *Bacteroides* and *Bifidobacterium*, induce mucosal glycan production, which further supports microbial growth, and the microbes convert indigestible milk glycans to absorbable short-chain fatty acids (55). He et al. (56) describe two different HMOs (3′-galactosyllactose and 2′-fucosyllactose) present in high amounts in colostrum. These HMOs affect pathogen-associated molecular pattern signaling pathways [Toll-like receptor (TLR) 3, TLR5, IL-1β, or cluster of differentiation 14 (CD14) expression and binding] decreasing cytokine production and the inflammatory response (56, 57). There are now even clinical trials examining the effect of probiotics added to the diet of lactating women, which increased the levels of secretory immunoglobulin A (sIgA) in the infant’s stool and on IL-6 mean values in colostrum and on IL-10 and TGF-β1 mean values in mature breast milk. This demonstrates the possibility of potentially beneficial microbes influencing the content of the woman’s breast milk and subsequently the infants’ GI tract *via* specific bioactive factors reaching the infant. The ultimate benefit to the infant remains to be studied (58). Human milk is not simply adding additional bacteria to the infant’s gut and intestinal microbiome but providing both bacteria and prebiotics to function in a symbiotic relationship creating the milieu in which the infant’s innate intestinal immunity functions and the intestine develops. How that symbiotic interaction influences the infant’s health in the future, as it relates to immune protection and immune reactivity (allergy or autoimmune disease) will require careful research involving not only genomics, metabolomics, proteomics, and glycomics but also epigenetics and techniques still need to be developed.

## Innate Mechanisms of Pathogen Recognition

There are specific TLRs present in human milk, including TLR2, TLR3, TLR5 as well as soluble CD14 (sCD14), and human β-defensin-1 (hBD-1), which function as pattern recognition receptors (PRRs) and AMPs (59). Chatterton et al. (59) discuss the potential role of PRRs in human milk affecting the protective response in the intestine balanced with other bioactive factors in milk affecting an anti-inflammatory mileau. There is evidence that TLR responses in the infant are modified by soluble TLRs (sTLRs) and sCD14. The interaction of both sTLRs and sCD14 with other bioactive factors in human milk upregulates and downregulates the action of various TLR-mediated inflammatory responses (60, 61). LeBouder et al. (61) demonstrated the effect of human milk on TLR-mediated microbial recognition. They describe specific responses on epithelial cells, monocytes, dendritic cells, and peripheral blood monocytic cells. The responses were different based on which TLRs were activated. Infant formulas did not exhibit such effects.

Immunoglobulins are the most recognized immune protective component in human breast milk. As preformed Igs from the mother, they constitute a discrete group of proteins capable of pathogen recognition. sIgA is the principal Ig in human milk (>90% of the Ig fraction), immunoglobulin M (IgM) in the pentameric form is next most abundant. There is a small amount of immunoglobulin G (IgG) in colostrum and transitional milk, with IgG becoming a much larger proportion of human milk Igs in mature milk (62). Secretory IgA binds pathogens blocking infection without stimulating a significant inflammatory response. In a largely innate immune-like action, sIgA simply blocks the pathogens contact with the intestinal epithelial layer and traps the pathogens within the mucin layers. The action of sIgA in the extracellular space is different from sIgA’s intracellular neutralization of viruses and bacterial lipopolysaccharides within epithelial cells. The glycan sugar component (galactose, fucose, and mannose) of sIgA contributes to sIgA resistance to proteolysis in the intestine and functions through a broad spectrum of binding of pathogenic bacteria when compared with the antigen specific binding of the variable region of the Ig structure. The broad spectrum binding related to the glycan sugar component of sIgA is more consistent with an innate immune response which would explain the absence of an increase in specific sIgA in the milk of mothers with sick infants (21).

Immunoglobulin M causes agglutination of recognized pathogens and complement activation as well as innate immune-like activities. Immunoglobulin G (IgG) activates phagocytosis with antigen transport to the lamina propria for B-cell activation affecting the infant’s adaptive response. The list of pathogens (viruses, bacteria, fungi, and parasites) recognized by human milk Igs is extensive (63). Gao et al. (64) report data from proteomic analysis of human milk, which demonstrate increased amounts of sIgA and IgM in transitional milk with IgG predominating in mature milk. They suggest that this transition fits with the infant’s developing immunity and evolving adaptive immunity to produce increasing amounts of IgG.

There are various other AMPs, in human milk, active in microbe killing, which can supplement the protection of the immature neonatal intestine (14, 65). hBD-1 is one example of an AMP in human milk, which affects pathogen membrane permeability and cytokine stimulation in the intestine (66). Other proteins [lysozyme, LF (and peptide derivatives of it—lactoferricin and lactoferrampin), α-lactalbumin, transferring, and osteopontin (OPN)] within human milk are recognized as important AMPs functioning *via* various mechanisms some of which enhance the anti-inflammatory effects of human milk (59, 64, 67). LF and its derivatives demonstrate a wide variety of actions on various targets including iron deprivation, destabilization of microbial membranes, binding microbial receptors, affecting chemokine production, stimulating epithelial cell growth, stimulating T-cell growth and differentiation, and production of reactive oxygen species (ROSs). LF also interacts with other components in human milk such as OPN, ceruloplasmin, and neutrophil peroxidase, although the exact significance and function of these interactions remain uncertain (22, 68). Xanthine oxidoreductase, another protein found in large amounts in milk and upregulated in mature milk, affects mammary epithelium, generation of milk fat droplet membranes, and adds to the bactericidal effect of human milk by synthesizing ROS (64, 66). These different AMPs do not simply act in microbial recognition and inactivation; each have different secondary functions within the intestine. Equally important is the limited inflammatory response generated by these AMPs in the gut.

## Anti-Inflammatory Factors and Effects

Maintenance of a homeostasis between protective inflammation and modulation of inflammation is essential to protecting the infant against infection at the same time as limiting the tissue damage due to inflammation (59). Oxidative stress through the production of free radicals does have some potentially beneficial effects for the host in terms of antibacterial action, immune defense, and signal transduction. The oxidative activity within the infant must be maintained in equilibrium with antioxidant capacity of tissues (69). Enterocytes and immune cells produce anti-inflammatory cytokines including transforming growth factor-beta (TGF-β), IL-10, IL-11, and IL-13. These factors act in an innate manner in the intestine.

Human milk caseins, LF, LP, OPN, Igs, superoxide dismutase (SOD), platelet-activating factor acetylhydrolase, and alkaline phosphatase each have both infection protective and anti-inflammatory effects. Specific hormones or growth factors predominantly exert their anti-inflammatory effects on intestinal innate immunity through their action on the proliferation and differentiation of IECs and immune cells (lymphocytes and macrophages) and modulating the inflammatory cytokine response. Transforming growth factors-β2 and -β1 upregulate tight junction proteins (caludin-1 and claudin-4) and downregulate TNF-α and IL-1β. In addition to growth stimulation, TGF-β has anti-inflammatory properties through stimulation of epithelial cell migration and repair of the epithelium after mucosal damage (70). Similarly, insulin-like growth factors, milk fat globule epidermal growth factor-8 (MFG-E8), and epidermal growth factor (EGF) influence growth and proliferation of IECs. Both MFG-E8 and EGF diminish the activation of nuclear factor kappa-light-chain enhancer of activated B cells (59). Trefoil factor 3 (TFF3) is an effector molecule that is present in the intestine and in large amounts in human breast milk. Generally, this molecule improves healing in the GI tract. The TFF3 present in breast milk produces downregulation of cytokines and promotes hBDs expression in IECs (71). Glucagon-like peptide 1 (GLP-1) is secreted from the enteroendocrine cell in the distal intestine and plays a role in regulating glucose metabolism and food intake. GLP-1 likely acts through vagal afferent pathway ultimately influencing feeding behavior (72). Recently, the first study to report GLP-1 in human milk showed that it was higher in hindmilk compared to foremilk and was correlated with infant weight gain during the first 6 months of life (73). Alternative forms of neonatal nutrition such as formula and TPN do not contain these anti-inflammatory properties, which may put these infants at a disadvantage by creating a relative deficiency of anti-inflammatory factors and activity.

Any enteral feeding directly stimulates growth of the neonatal gut. Human milk contains specific direct growth factors including platelet-derived growth factor, hepatocyte growth factor (HGF), vascular endothelial growth factor, and insulin (74–76). Each of these is important in angiogenesis, cell development, and tissue proliferation. HGF is expressed in the intestinal tissues and is present in human milk. HGF may play a role in mucosal growth and repair (77). Animal studies have shown that HGF given after intestinal resection or colitis has improved gut proliferation and nutrient transport (78, 79). Weiss et al. (80) describe declining levels of anti-inflammatory, proresolving lipoxin A4 (LXA4), and resolvin D1 (RvD1) and D2 (RvD2) in the lipid profile of human milk from colostrum through the first month of life. The average amount of LXA4 in human milk was two times the amount of proinflammatory leukotriene B4 (LTB4) (80). This highlights the importance of minimizing inflammation in the early period of an infant’s life.

Specific antioxidants in human milk include vitamins A, E, C, LF, lysozyme, glutathione peroxidase, SOD, catalase, ceruloplasmin, coenzyme Q10, thioredoxin, leptin, adiponectin, and trace elements—iron, copper, zinc, and selenium (69). These antioxidants act by reacting directly with a free radical before damage occurs or by interfering with the ongoing oxidation in liquid phase or in cell membranes. The total antioxidative capacity of human milk is highest in colostrum and declines over lactation with variability from person to person and time to time (69). Adiponectin, leptin, LF, and lysozyme each have antioxidant effects, and their concentrations in human milk also vary over time (81). Vitamin A or its derivatives bind to radicals of oxygen, thiol, or peroxide, limiting their oxidative damage on cells. There are higher concentrations of vitamin A in colostrum than mature milk (82). Higher concentrations of α-tocopherol (the predominant form of vitamin E) are in colostrum than transitional or mature milk (83). Vitamin E forms part of the milk fat globule membrane and constitutes the major portion of antioxidative function of breast milk at 1 month of age (84). Vitamin C is a hydrosoluble vitamin in human milk and an effective antioxidant in extracellular fluids.

Nucleotides, nucleosides, and nucleic acids are essential to cellular metabolic function. They constitute approximately 15–20% of the non-protein nitrogen or total potentially available nucleosides in human milk (85). Nucleotides function predominantly in cellular energy metabolism related to adenosine triphosphate, as messengers and coenzymes in metabolic pathways, and in nucleic acid production and salvage. In the face of infection, nucleotides are essential to the immune response (cellular activation, proliferation and action, and cellular signal transduction) and the repair of intestinal inflammation and damage. Brunser et al. (86) reported on the benefit of nucleotide supplementation for infants less than 6 months old at decreasing the severity and incidence of diarrhea. They postulated that the effect was due to effects on the intestinal integrity and repair as an example of an anti-inflammatory effect.

## Evolution of Bioactive Factors in Human Milk Over Lactation

The composition of human milk is dynamic with significant change from colostrum, transitional to mature milk, between preterm and term milk and with interindividual and intraindividual variation. Specifically, HMOs show interindividual variation relative to the total number of HMOs and individual HMOs varying with mother’s Lewis blood group and secretor status.

Colostrum is produced between birth through the first 5 days of lactation, transitional milk is from 5 days to 2 weeks postpartum and maturation of the milk continues until it is “fully mature” at 4–6 weeks postpartum. There is only a small volume of colostrum produced, rich in leukocytes, protein, HMOs, and bioactive factors—IgA, LF, EGF, TGF-β, colony-stimulating growth factor, and antioxidants (31). Transitional milk has decreasing amounts of protein and Igs and increasing lactose and fat and water-soluble vitamins resulting in a higher caloric density of the milk to meet the infant’s growth demands while the quantities of bioactive factors declines over time. The composition of mature milk remains constant after 6 weeks through the remainder of the lactation period. The amount of Igs and LF in milk decreases over the first 3–4 months, while the amount of lysozyme increases (87). Tregoat et al. (88) described declining amounts of mannan-binding lectin concentrations in transition from colostrum to mature milk. More recent published studies using proteomic analysis of human milk (16, 64, 89) continue to facilitate our understanding of the complex nature of human milk and its role related to immune function and intestinal development. Gao et al. (64) describe a similar transition of the various Igs, from colostrum to mature milk using proteomic analysis of the milk. They describe a large percentage of milk proteins having to do with immune function including complement factors and serine protease inhibitors important in regulating the complement system. The quantities of these factors declined in transition from colostrum to mature milk. They also identified proteins associated with the extracellular matrix including cytokines, fibronectin, tenascin, and OPN. These proteins were more prevalent in transitional milk. Glutathione and antioxidant activity-related proteins were more common in mature milk. Overall, the quantity of immune factors and immune effects of human milk diminish over time in parallel with the developing immune system of the infant. Nevertheless, it will be essential to understand the specific roles of the various bioactive components of human milk and how the change in milk composition over time influences the evolving effects of human milk on the intestine and innate immunity.

## Differences in Preterm and Term Human Milk

Preterm babies require additional nutrition and immune protection compared to term infants. Interestingly, preterm breast milk has been found to contain increased nutrients such as protein (90) and higher concentrations of certain immune factors. Preterm human milk also has higher amounts of phagocytes and secretory IgA (24). These increased amounts may serve a protective role since premature infants have poorly functioning neutrophils, limited production of Igs, and lower levels of passively acquired Igs.

A few studies reported a difference in breast milk microbiome when comparing preterm and term milk. Some trends include more *Bifidobacterium* in term milk (91) and more Enterococcus in preterm milk (91). A study looking at preterm infants, testing stool and breast milk samples, found a high proportion of antibiotic-resistant high-risk clones in both fecal and milk samples during the neonatal intensive care unit (NICU) admission (92). Differences are also seen in gut microbiome between term and preterm infants. Variations in microbiota of preterm infants have been described as predisposing to development of necrotizing enterocolitis (NEC) (93–97). Hormones and cytokines also vary by gestational age. EGF has anti-inflammatory properties and is higher in preterm milk compared to full-term milk (24) The cytokine IL-10, with anti-inflammatory properties, was detected in lower amounts in breast milk for infants with increased risk of NEC (98). Trend et al. reported that TGF-β2 concentrations in human milk were significantly higher in the extreme premature infant group compared to the term infant group (99). Castellote et al. also reported that TGF-β2 was higher in preterm infants compared with term infants (100). A study by Maheshwari et al. demonstrated that this anti-inflammatory cytokine suppresses endotoxin-induced cytokine responses of gut macrophages in the preterm infant *in vitro* and protects rat pups from gut injury *in vivo* (101). They also reported that percentages of IL-10 and TNF-α were lower in preterm milk compared to term milk.

Trend et al. found that AMPs can limit the *in vitro* growth of bacteria associated with neonatal sepsis (52). This is particularly important in preterm infants who are at increased risk of late-onset sepsis. The hBD-1 was higher in preterm colostrum compared to term colostrum (99). Wang et al. (102) also found that hBD-1 and hBD-2 were in higher concentration in mature preterm milk than mature term milk. Armogida et al. and Trend et al. both found that human α-defensin 5 levels were not affected by preterm birth, which suggests that these defensins are differentially regulated (99, 103). The amount of LF in human milk does not seem to be affected by gestational age (99) but is more dependent on milk volume expressed. There are conflicting results for concentrations of lysozyme in preterm and term milk.

It has been proposed that the increased levels of immune factors in preterm milk may be a result of a compensatory mechanism whereby in the mother during preterm labor, the breast shifts the immune content of the milk to provide more protection. Another explanation suggested by Goldman et al. (62) is that increased immune factors in preterm milk may be due to increased maternal systemic inflammation, a postulated condition leading to preterm delivery. The mechanisms for the differences between preterm and term milk remain unknown. Interestingly, most immune factors decrease over the first month regardless of gestational age, thus term and preterm milk become more similar over time as the chronological age of the baby increases.

## Summary

Feeding an infant human breast milk is not a matter of filling the infant with an “appropriate” amount of important nutrients and a protective level of bioactive factors. Although the various factors do complement and supplement the innate immunity of the infant, they actively affect the ongoing development of the infant’s immunity and intestinal development. As Lars Bode et al. declared, “Human milk is alive, …” (27). The cells, the microbes, and the bioactive factors make milk alive, and the interactions of human milk with its natural host, the infant, create a symbiotic commensal relationship. This is the challenge to explain and understand the complexity and dynamic relationship between the everchanging secretion, human breast milk and the developing, evolving human infant.

## Author Contributions

All authors have made substantial, direct, and intellectual contribution to the work and approved it for publication. NC is primarily responsible for Figure 1.

## Conflict of Interest Statement

The authors declare that the research was conducted in the absence of any commercial or financial relationships that could be construed as a potential conflict of interest.
